# Rabies elimination research: juxtaposing optimism, pragmatism and realism

**DOI:** 10.1098/rspb.2017.1880

**Published:** 2017-12-20

**Authors:** Sarah Cleaveland, Katie Hampson

**Affiliations:** Institute of Biodiversity, Animal Health and Comparative Medicine, College of Medical, Veterinary and Life Sciences, University of Glasgow, Glasgow G12 8QQ, UK

**Keywords:** rabies, elimination, dog vaccination, One Health, neglected tropical disease

## Abstract

More than 100 years of research has now been conducted into the prevention, control and elimination of rabies with safe and highly efficacious vaccines developed for use in human and animal populations. Domestic dogs are a major reservoir for rabies, and although considerable advances have been made towards the elimination and control of canine rabies in many parts of the world, the disease continues to kill tens of thousands of people every year in Africa and Asia. Policy efforts are now being directed towards a global target of zero human deaths from dog-mediated rabies by 2030 and the global elimination of canine rabies. Here we demonstrate how research provides a cause for optimism as to the feasibility of these goals through strategies based around mass dog vaccination. We summarize some of the pragmatic insights generated from rabies epidemiology and dog ecology research that can improve the design of dog vaccination strategies in low- and middle-income countries and which should encourage implementation without further delay. We also highlight the need for realism in reaching the feasible, although technically more difficult and longer-term goal of global elimination of canine rabies. Finally, we discuss how research on rabies has broader relevance to the control and elimination of a suite of diseases of current concern to human and animal health, providing an exemplar of the value of a ‘One Health’ approach.

## 1. Introduction

For thousands of years, people have lived in fear of rabies transmitted from domestic dogs, and more than half of the world's population still do so today. From the time of the first written reference to rabies in the 23rd century BC, the link between the bite of a mad dog and the risk of human death has been well recognized [1,2]. Although many mammalian hosts can be infected with the rabies virus, the domestic dog remains to this day by far the most important species causing human rabies deaths and tens of thousands of people die from canine-mediated rabies each year [3,4], mostly in Asia and Africa where the disease is maintained in domestic dog reservoirs.

In developing the first vaccines against rabies, Louis Pasteur recognized the potential for eliminating human rabies deaths, and considered that ‘to solve the problem of rabies would be a blessing for humanity’ [5]. The need for and feasibility of rabies elimination through interventions in the dog population has also been recognized for more than a century. Since the first large-scale implementation of canine vaccination in the 1920s, canine rabies has now been eliminated in several parts of the world, for example in island and peninsula states of Asia (e.g. Japan, Taiwan), in the USA, western Europe and across parts of Latin America [1,6,7].

In this review, we address the reasons why, despite the long history of rabies research and earlier successes in canine rabies elimination, new research has been needed to tackle the problem of rabies in low- and middle-income countries (LMICs) of Africa and Asia. We demonstrate how research has generated optimism about the feasibility of achieving global targets of zero human deaths from dog-mediated rabies, guided pragmatism in the design of dog vaccination strategies in LMICs, and instilled realism in the path towards global canine rabies elimination.

## 2. Shifting priorities in rabies research

**Figure 1.**
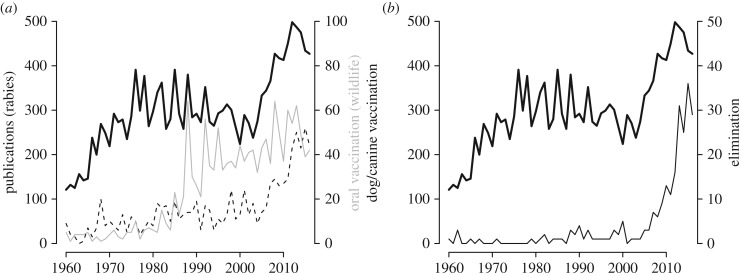
Number of journal articles published on rabies from 1960 to 2016 illustrating: (*a*) the shift in emphasis from oral vaccination of wildlife in the 1980s and 1990s to dog vaccination from 2000s; and (*b*) the increase in publications relating to canine rabies elimination since 2010. A search on Web of Knowledge was used to identify journal articles: (*a*) articles with rabies in the title (solid black line) and of these, articles referring to oral vaccination of wildlife (solid grey line) and canine/dog vaccination (dotted black line); and (*b*) articles with rabies in the title (solid black line) and, of these, articles relating to canine/dog rabies elimination (dark grey line). Further details are provided in the electronic supplementary material. Note that dog rabies and dog rabies elimination are plotted on different axes.

While the first decades of rabies research focused on the problem in domestic dogs, the successful control of canine rabies in many of the world's richer countries shifted emphasis towards the growing problem of wildlife rabies. During World War II, the red fox (*Vulpes vulpes*) emerged as the main rabies reservoir in Europe, and the disease spread rapidly affecting most of western and southeastern Europe by the mid-1970s [8]. In response, rabies research efforts focused on development of oral rabies vaccines and vaccination strategies for wildlife (figure 1), with large-scale distribution of oral bait vaccines across western Europe in the 1980s and 1990s [8]. Over 25 years, oral vaccination of foxes has resulted in the elimination of the rabies virus from western Europe, with rapid progress being made towards elimination in eastern Europe [9].

Over this same time period, canine rabies was being brought under control in north America, and research efforts independently became directed to the emerging problem of wildlife rabies focusing on control of rabies in terrestrial carnivore reservoirs [10]. A further concern in north America related to bat-transmitted rabies [11], coinciding with a growing interest in bats as hosts of a wider range of Lyssaviruses, [12] and other emerging pathogens of global concern, such as SARS coronavirus, Ebola virus and MERS coronavirus.

It is not surprising that set against this backdrop, research into the control of canine rabies in LMICs received only limited attention during the latter part of the twentieth century (figure 1). However, this resulted in a deficit of data and understanding of the burden and scale of the disease in poorer parts of the world and limited interest in potential solutions, reinforcing a cycle of neglect [13].

## 3. Insights from studies on the global burden of canine rabies

It has always been known that dog bites are an important source of human rabies exposures worldwide, but reliable data have been lacking on the number of dog-mediated human rabies deaths [14], with the few hundred deaths officially reported in the African region [15] widely recognized to be a major underestimate.

An initial approach to estimating human rabies deaths in Africa used a probability decision tree model that incorporated data on the incidence of bite injuries from suspected rabid dogs and availability of post-exposure prophylaxis (PEP) [16]. This was first applied in Tanzania and then used to generate country- and regional-level estimates of human deaths across Africa and Asia [17–21] and to assess the economic impacts of canine rabies [22]. Further refinements resulted in more detailed and comprehensive estimates of global disease burden by country [4]. These studies indicated that more than 99% of canine-mediated rabies deaths occurred in Africa and Asia, with a global estimate of 59 000 (95% confidence interval (CI) 25 000–159 000) deaths in 2010 [4]. Other approaches have been adopted by the Global Burden of Disease (GBD) collaborators, including a cause of death ensemble modelling approach, which have generated estimates ranging from 54 100 deaths (95% CI 32 400–103 400) in 1990, 26 400 (95% CI 15 200–45 200) in 2010, 23 500 (95% CI 17 300–28 600) in 2013, and 13 300 in 2016 (95% CI 7200–19 100) [3,23,24].

It is well recognized that these modelling approaches all have limitations, particularly in the degree of extrapolation from data that is of variable quality, from a limited geographical area or that has been generated indirectly [25]. For several neglected tropical diseases (NTDs), GBD figures are thought likely to represent an underestimate of current disease burden [26]. For rabies, there is no evidence that control measures have been implemented on a scale that would explain the dramatic recent decline in deaths indicated by the GBD estimates [23,24]. GBD estimates rely on vital registration and verbal autopsy data and these are very limited or absent in many of the countries where rabies and other NTDs are most prevalent [24]. Another critical issue is the appropriate modelling of pathways from infection to disease and death [26]. While the rabies probability tree study [4] was also limited by data quality and availability, this analysis incorporated detailed data from disease-specific research in rabies-endemic countries and was based on a well-defined series of steps from rabies exposure to death. We draw further confidence from comparison with estimates derived from empirical studies. For example, annual human rabies deaths in India were estimated at 20 565 through a multi-centre community survey conducted in 2003 [27], very similar to the figure of 20 847 deaths derived for India in 2010 through the probability tree approach [4].

**Figure 2.**
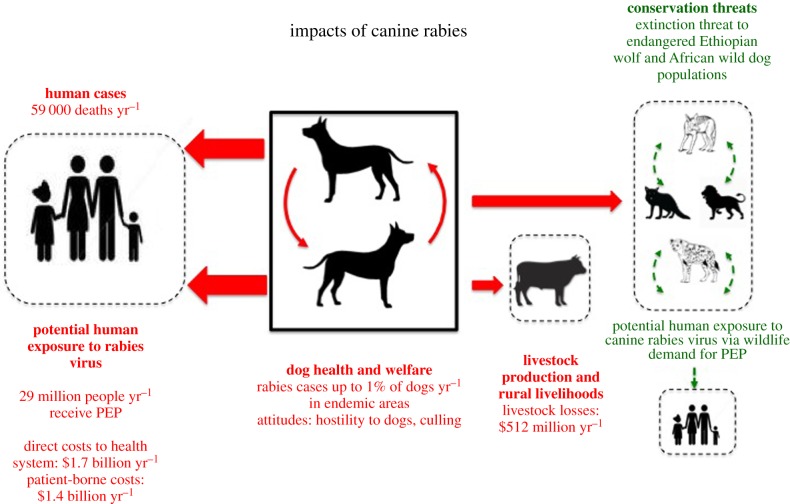
Schematic illustrating the impacts of canine rabies and role of domestic dogs in maintaining rabies transmission (shown by solid lines). Other host species (humans, livestock and wildlife) may be infected as a result of spillover transmission from dogs but cannot sustain cycles of infection independently (shown by dotted lines). With the control and elimination of rabies in dogs, the virus is likely to disappear in all other species, with the potential for benefits to human, domestic animal and wildlife health, and substantial cost savings. (Online version in colour.)

The PEP data used in the probability tree model also provided important information for demonstrating the economic burden of canine rabies, indicating that $1.7 billion direct costs were incurred annually in providing PEP for 29 million dog-bite victims in canine-endemic countries [4] (figure 2). Regionally, the highest expenditure is seen in Asia ($1.4 billion annually) reflecting a continuing high demand for PEP in areas where canine rabies has not been brought under control, and contrasting with Latin America where, despite much lower annual expenditure on PEP ($129 million), the region is on the brink of eliminating canine-mediated human rabies as a result of relatively modest investments in mass dog vaccination ($61 million) [4]. These data contribute to a growing body of evidence that the most cost-effective preventive strategies are those underpinned by mass dog vaccination rather than reliance on PEP alone [28–30].

Compiling data for the global burden study also highlighted PEP availability as a major determinant of human rabies deaths, with cases occurring disproportionately in impoverished rural communities. Detailed contact tracing studies reveal the extent to which people have struggled to obtain PEP and the consequences of the resulting delays [31,32], which invariably include intense anxiety as bite victims await an uncertain outcome and, in some cases, the development of a horrifying and fatal disease.

While human deaths and high PEP costs dominate in burden of disease studies, several other components of disease burden are also of concern, including livestock losses, which still remain poorly quantified but can have important impacts [22,33] and wildlife conservation, with canine rabies threatening several endangered wildlife populations including the Ethiopian wolf (*Canis simensis*) and African wild dog (*Lycaon pictus*) [34] (figure 2).

## 4. The feasibility of canine rabies elimination: a cause for optimism

A considerable body of research now exists to demonstrate the feasibility of canine rabies elimination. The basic reproductive number, *R*_0_, a key parameter used to understand the effectiveness of control interventions, is usually measured from the growth rates of epidemics. Applying this approach to canine rabies demonstrates that *R*_0_ is typically between 1 and 2 in populations that differ in density by an order of magnitude [35–37]. Alternative approaches to estimating transmission are all consistent with this low value of *R*_0_ [29,36,38,39] suggesting that rabies should be easily controlled through mass dog vaccination and, conversely, that approaches based on reducing dog density are likely to be ineffective [40]. Theoretical and empirical research has demonstrated that rabies can be eliminated where 70% coverage is sustained [35,36]. By contrast, attempts to reduce dog population density through indiscriminate culling have consistently failed to control rabies outbreaks [41] and, in some cases, have increased disease spread through human-mediated dog movements [37]. Muzzling, restriction of dog movements, and selective removal or euthanasia of unowned dogs have historically been part of successful dog rabies control, including in the UK and USA [1,42], but these measures are distinguished from indiscriminate culling operations in being specifically targeted to reduce rabies transmission risk rather than to reduce dog population size or density.

The question of rabies reservoir dynamics has long been debated [6,43–47], and is of major importance in sub-Saharan Africa where the abundance of wildlife has been seen as an obstacle for canine rabies control that would render elimination efforts futile [15]. However, despite the fact that rabies can infect all mammalian species, only a few hosts are capable of maintaining infection as reservoirs, with ecological and genetic factors both likely to be important determinants of rabies reservoirs [46,47]. While rabies virus variants are typically maintained by only a single mammalian host species, multiple variants may circulate in an area [47]. However, this need not be an insurmountable obstacle to canine rabies elimination, as shown by countries in Latin America and in the USA, where canine rabies has been brought under control or eliminated even though rabies variants circulate in wild mammal populations. The overlapping circulation of multiple variants does, however, introduce different surveillance requirements for verifying the elimination of the canine rabies variant.

Establishing the reservoir of multi host pathogens is not easy and typically requires integration of multiple lines of evidence [44,48]. In the Serengeti ecosystem, Tanzania, inference from both epidemiological and genetic data supports the idea of rabies being maintained in domestic dogs not wildlife, with occasional spillover from domestic dogs into wildlife resulting in short-lived chains of infection that are not sustained [49–51]. The conclusion from these studies is that control of canine rabies should eliminate infection in dogs, wildlife and people. It is unclear the extent to which the Serengeti scenario is generalizable more globally, but currently there is no clear evidence that, in areas with domestic dog reservoirs, the canine rabies virus variants circulating in dogs are maintained independently in wildlife. In South Africa, a canine variant circulates in jackals in the Limpopo region [52], but it is still unknown whether this cycle will be sustained in the absence of canine rabies, which has now been well controlled in the area. If so, vaccination of jackals may be needed to achieve canine rabies elimination, but this is likely to be feasible given the demonstration of the safety, efficacy and feasibility of oral vaccination in jackals from earlier work in Zimbabwe and Israel [53–56].

Demonstration of the operational feasibility of mass dog vaccination provides a further cause for optimism. Evidence now exists to show that, contrary to widely held perceptions, the vast majority of dogs in Africa have owners, dog accessibility is higher than often recognized, and achieving target levels of vaccination coverage is feasible [57,58]. In south and southeast Asia, the situation may be more challenging as a result of a larger population of less accessible community or ‘street’ dogs, but target levels of vaccination coverage have also been achieved in these communities where campaigns are well organized [37,59,60].

In summary, the last decade has seen a rapid expansion of research into canine rabies vaccination and canine rabies elimination (figure 1) generating optimism that canine rabies can be effectively controlled, and ultimately eliminated, through mass dog vaccination and that this is the underpinning strategy needed to reach the 2030 target for elimination of human deaths from canine-mediated rabies [61]. The health and economic benefits would be substantial [22] (figure 2).

## 5. Strategies for control and elimination of canine rabies: a case for pragmatism

While it is often recommended that a detailed understanding of dog ecology is needed for effective canine rabies control, the consistency of research findings generated over the past 30 years allows us to be confident in concluding that mass dog vaccination is feasible across a wide range of settings and campaigns can and should be initiated without delay. In some cases, more nuanced understanding may be required to improve coverage, but these insights can be often be gained through implementation of control measures and used to progressively improve the design and delivery of subsequent interventions. Key considerations include the nature and degree of community engagement, timing of campaigns, placement of vaccination stations and whether or not to charge owner fees [62–64]. The costs of implementing campaigns free of charge may exceed those readily available to government veterinary services [65], but many approaches can still be explored to improve affordability, acceptability and cost-effectiveness [66].

While there is widespread agreement about the central importance of mass dog vaccination in canine rabies control and elimination, the role of dog population management remains the subject of debate [67]. There is a rich literature around fertility control for management of roaming dog and wildlife populations [68,69]. However, as rabies transmission varies little with dog density, reproductive control measures carried out with the aim of reducing dog density are not likely to be effective for rabies control. In theory, reducing population turnover (e.g. through improving life expectancy and/or reducing fecundity) could help sustain population immunity between campaigns and improve cost-effectiveness. However, there is little empirical evidence that dog population management tools have been able to achieve this [67]. Furthermore, even in populations with a high turnover, achieving a 70% coverage during annual campaigns has been sufficient to sustain population immunity above critical thresholds determined by *R*_0_ [70]. The relatively high cost of sterilization also means that strategies which combine vaccination and sterilization are less cost-effective in terms of achieving human health outcomes than strategies based on dog vaccination alone, even in populations with a large proportion of roaming dogs [39]. Improved dog population management is undoubtedly a desirable longer-term goal for animal health and welfare and may have important secondary benefits for rabies control, for example by enhancing community or political support [67]. However, a focus on mass dog vaccination currently remains the most pragmatic and cost-effective approach to canine rabies control and elimination.

The limited availability and quality of routine animal rabies surveillance data in LMICs [14] has been an obstacle to the application of the analytical approaches from which we have learned so much about wildlife rabies. ‘Gold standard’ surveillance data based on laboratory-confirmed diagnosis is hampered not only by limited laboratory infrastructure but also by the practical challenges of locating, sampling and submitting specimens [71]. However, pragmatic approaches to improving rabies surveillance have yielded rich insights. In addition to providing a foundation for burden of disease estimates, data on animal-bite injuries have been a used as a reliable indicator of canine rabies incidence, revealing new understanding of rabies metapopulation dynamics [50], as well as improving detection of animal rabies cases, the management of animal bites and the cost-effectiveness of PEP [36,72].

Pragmatic solutions are also being found to improve rabies diagnosis in settings with limited laboratory infrastructure, including techniques to support decentralized laboratory testing (e.g. direct rapid immunohistochemical test, dRIT) [73–76] and field diagnosis (e.g. immunochromatographic tests) [77–79]. These have great potential for empowering field staff to engage in rabies surveillance and respond more effectively to surveillance data, but standardization and quality control of field diagnostic kits still needs improvement [80]. Given the rapid advances in metagenomic sequencing methods [81], future approaches may include real-time genomic surveillance. However, even simple technologies such as mobile phones can serve as leapfrogging technology that can dramatically improve the extent and resolution of rabies surveillance data [82].

## 6. Instilling realism on the path to elimination

**Figure 3.**
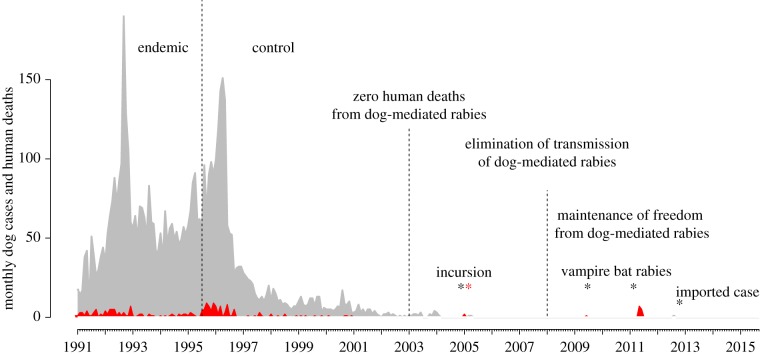
Hypothetical timeline of rabies control and elimination highlighting policy targets and epidemiological milestones, illustrating relative rapid progress to zero human deaths but the need for sustained effort to reach elimination of canine rabies and sustained surveillance to identify the causes of cases. In this example, drawn from a scenario typical of Latin America, human cases following declaration of zero human deaths from dog-mediated rabies might occur as a result of (i) an incursion of canine rabies (in which case the rabies-free status of the country would be reset); and (ii) vampire bat rabies and (iii) an imported human case (in which cases the status of the country as being free of dog-mediated rabies would not change). Cases of canine rabies are shown in grey and human cases in red.

While operational research on dog vaccination provides grounds for optimism, awareness is growing about the challenges, complexities and time scales of moving from control to elimination (figure 3).

Given the low *R*_0_ for rabies, deterministic models of transmission predict that rabies should be eliminated very rapidly [29,30,83–85]. But, these dynamic models typically assume that dog vaccination campaigns consistently achieve high and uniform levels of coverage. By contrast, analyses of rabies surveillance and control data indicate that vaccination coverage implemented during campaigns is often patchy and that time to rabies elimination is prolonged [37,86,87]. Once assumptions about the implementation of vaccination campaigns are more realistic, and rabies is considered on a spatial landscape, predictions about the time scale to elimination are tempered [86].

The disparities between theory and practice demand approaches that capture realism. It may be argued that the feasibility and effectiveness of mass dog vaccination should have been self-evident given the successes in Latin America but the road to elimination has been accompanied by substantial challenges [88]. Progress in Latin America has required decades of investment in large-scale dog vaccination programmes and builds on effective regional coordination. Sustaining such coverage, particularly across large geographical areas, is difficult and requires an investment in rabies control that focuses on the dog population and is over and above levels seen to date in Africa and Asia [65]. Local leadership is also an important factor. For example, canine rabies in north America was primarily controlled at the municipal level through dog licensure. Legislation and by-laws relating to rabies control and dog vaccination exist in many canine rabies-endemic countries, but there is still a need for greater engagement of local authorities to ensure appropriate and sensitive enforcement of relevant legislation.

Empirical evidence from wildlife rabies elimination programmes show that once controlled to less than 10% of endemic incidence, the time required to eliminate rabies is as long again [9], a lesson that should be heeded for canine rabies. Once rabies has been reduced to low levels, the remaining foci by their nature are persistent and in ‘hard-to-reach’ communities, socially, economically and geographically, and new challenges come to the fore [89].

Increasingly the importance of metapopulations has been recognized for the persistence of rabies [38,50] and genomic signatures in rabies-endemic countries highlight the frequent human-mediated movement of dogs [90–92]. The implications of this movement are evident when rabies invades previously uninfected areas [37], and without maintained vigilance, rabies can re-emerge rapidly if control measures are no longer implemented effectively [93]. The long-term implications of these incursions to the persistence of rabies are not yet fully understood but will, undoubtedly prolong elimination efforts, and highlight the need for coordinated control at scale and across international boundaries as well as realistic projections of the investment required to eliminate rabies [94].

## 7. Rabies research as a driver of policy change

Recent research has contributed pivotal evidence in making the case for rabies to be considered a priority NTD and, in 2012, rabies was included within the World Health Organization (WHO) Accelerated Roadmap for NTDs [95]. In 2016, the tripartite partnership (WHO, the World Organization for Animal Health (OIE), and the Food and Agriculture Organization of the United Nations (FAO), together with the Global Alliance for Rabies Control, declared a goal of zero human deaths from dog-mediated rabies by 2030 [61], underpinned by an investment case incorporating data on the human health and economic burden of canine rabies [4,22]. This purposely sets dog-mediated human rabies deaths as the first target, both because of its public health importance, but also its shorter-term feasibility (figure 3) through a combination of mass dog vaccination and improved PEP provision to under-served communities. The longer-term goal of disrupting transmission and eliminating canine rabies will require more time. Nonetheless, the example of Latin America demonstrates that it is within reach [88].

The control and elimination of canine rabies provides an exemplar of ‘One Health’ interventions, that is, interventions in animal populations that generate human health benefits. Although challenges remain in the operationalization of One Health [96], these approaches not only provide the most cost-effective strategy for preventing human rabies deaths but also offer a more equitable approach than relying only on interventions directed at humans only (i.e. PEP) [97]. Interventions that effectively reduce the force of infection from the animal reservoir convey benefits to all without regard to socioeconomic status. By contrast, under a strategy of reliance on PEP, the social, political and economic factors constraining access to healthcare are likely to prevail, with rabies deaths continuing to affect the most disadvantaged communities well beyond 2030.

It is perhaps understandable that the medical sector emphasizes prevention of human rabies through PEP. But this approach can lead to neglect of the problem at source—in the dogs—and impede progress towards large-scale mass dog vaccination programmes. This is true even in upper middle-income countries which have clear capability to implement mass dog vaccination but, without effective programmes, still suffer a high burden of human deaths and an escalation in PEP demand, with costs amounting to tens of millions of dollars every year [4].

## 8. Conclusion

Recent research on rabies has generated a strong body of evidence for the feasibility of elimination of canine rabies through mass vaccination of domestic dogs. Global momentum is now building towards implementation of large-scale programmes to achieve first, the elimination of human deaths mediated by canine rabies, and second, disruption of transmission within the dog population and the elimination of canine rabies entirely. However, time is short to reach these global targets [61] and there is no cause for further delay.

## Supplementary Material

File S1

## Supplementary Material

Data on rabies publications

## Data accessibility

Data relating to the number of published articles shown in figure 1 are provided in the electronic supplementary material.
