# Supplementary material for: Rabies elimination research: juxtaposing optimism, pragmatism and realism
Source: Proc Biol Sci. 2017 Dec 20;284(1869):20171880. doi: 10.1098/rspb.2017.1880 (PMC5745407; doi:10.1098/rspb.2017.1880)
Supplement: File S1 [file rspb20171880supp1.docx]

**File S1**

**Fig 1: Protocol for search of articles on rabies**

**Search strategy:** A database search was performed on Web of Science for resources published between 1^st^ January 1960 and 31^st^ December 2016. We searched for all rabies publications using Search box 1: Title = (Rabies). For publications relating to oral vaccination, we used search box 1 AND search box: Topic = (oral vaccination OR oral immuni*). For publications on dog vaccination, we used search box 1 AND search box: Topic =(canine OR dog) AND Topic = (vaccination OR immuni*). For publications on canine rabies elimination, we used search box 1 AND search box: Topic = (elimination) AND Topic = (dog OR canine).

Titles were extracted into Excel together with information on authors, article type, year of publication, volume, issue and page numbers. Further steps included selection of journal articles only (PT=J) and removal of Figshare references. Articles were sorted by title, followed by authors and year of publication for manual de-duplication. Articles were marked for removal if sharing the same authors, the identical title, volume number and year of publication. Information on issue and page numbers was not available for all articles. To select for articles addressing oral vaccination of wildlife rather than dogs, titles containing the term ‘dog’ were identified and were marked for exclusion if referring only to dogs in the title (and not raccoon dogs, prairie dogs or African wild dogs) and with no reference to wildlife. To select for articles on dog vaccination, titles were searched to exclude those making reference to raccoon dogs, prairie dogs or African wild dogs, without any reference to vaccination of domestic dogs.

Search 1. Rabies (title): Total journal articles = 17961; total after removal of duplicates references = 17126

Search 2. Rabies (title) AND oral vaccination/immunization of wildlife (topic): Total journal articles = 1539; total after removal of duplicates = 1493; total after removal of articles referring only to dogs in the title with no reference to wildlife = 1414.

Search 3. Rabies (title) AND dog/canine vaccination/immunization (topic): Total journal articles = 981; total after removal of duplicates = 936; total after removal of articles referring to wildlife in the title (raccoon dogs, prairie dogs or African wild dogs) =895

Search 4. Rabies (title) AND canine/dog elimination (topic): Total journal articles = 252; total after removal of duplicates = 234.
